# Cyanoacrylate injection for hemorrhagic giant esophageal varices: simultaneous disappearance of the varices with hemostasis

**DOI:** 10.1055/a-2436-1412

**Published:** 2024-10-25

**Authors:** Kazunori Nagashima, Yasunori Inaba, Ken Kashima, Yasuhito Kunogi, Fumi Sakuma, Akira Yamamiya, Atsushi Irisawa

**Affiliations:** 1 Department of Gastroenterology, Dokkyo Medical University School of Medicine, Shimotsuga, Japan

Cyanoacrylate injection for hemorrhagic gastric varices is safe and effective, providing high technical success 1 . Endoscopic variceal ligation (EVL) is usually performed for hemorrhagic esophageal varices 2 . However, giant varices without palisade vessels (i.e. pipeline varices) are difficult to treat 3 . To the best of our knowledge, this report is the first video case of endoscopic injection with cyanoacrylate for giant pipeline esophageal varices, resulting in hemostasis and simultaneous disappearance of the varices themselves.

This video presents a typical case ( Video 1 ). The patient, a 67-year-old man, had alcoholic cirrhosis and giant esophageal varices ( Fig. 1 ), which were scheduled for hemorrhage prevention treatment. He had been rushed to the emergency room earlier for varix bleeding. Three-dimensional contrast-enhanced computed tomography (3D-CT) showed the hemodynamics of the pipeline varix, which was fed from the left gastric vein ( Fig. 2 ). After attempting banding on the bleeding point, which was unsuccessful because of the large varices ( Fig. 3 ), we used compression hemostasis using a balloon. After cyanoacrylate was injected into the gastric side of the bleeding point to the blood supply route ( Fig. 4 ), complete hemostasis was achieved; furthermore, the varices themselves disappeared ( Fig. 5 ).

**Fig. 1:**
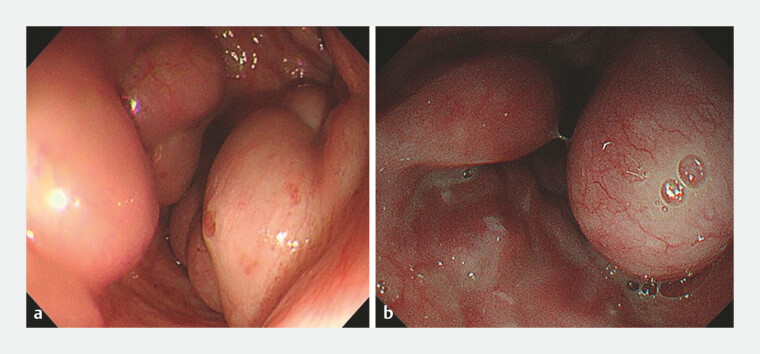
The varices were large and showed strong development.

**Fig. 2:**
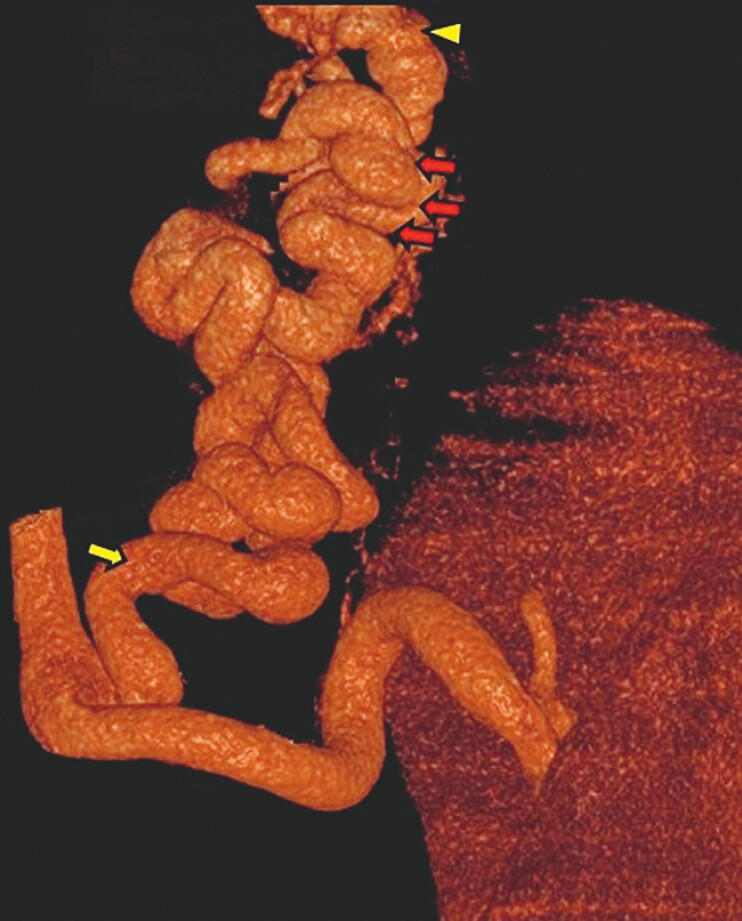
Three-dimensional computed tomography revealed the hemodynamics of the pipeline varix (red arrows), which was fed from the left gastric vein (yellow arrow) to the azygos vein (yellow arrowhead).

**Fig. 3:**
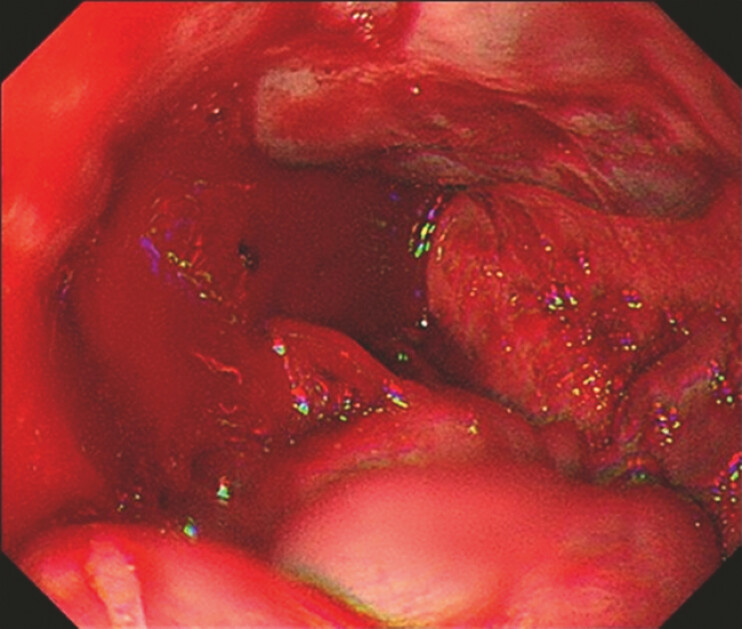
The bleeding varix point was found at the 6 o’clock position.

**Fig. 4:**
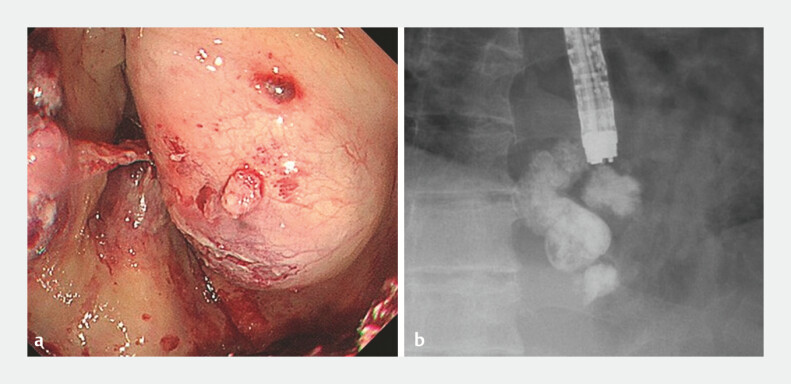
Cyanoacrylate was injected into the varices on the gastric side of the bleeding point. The feeder was also occluded.

**Fig. 5:**
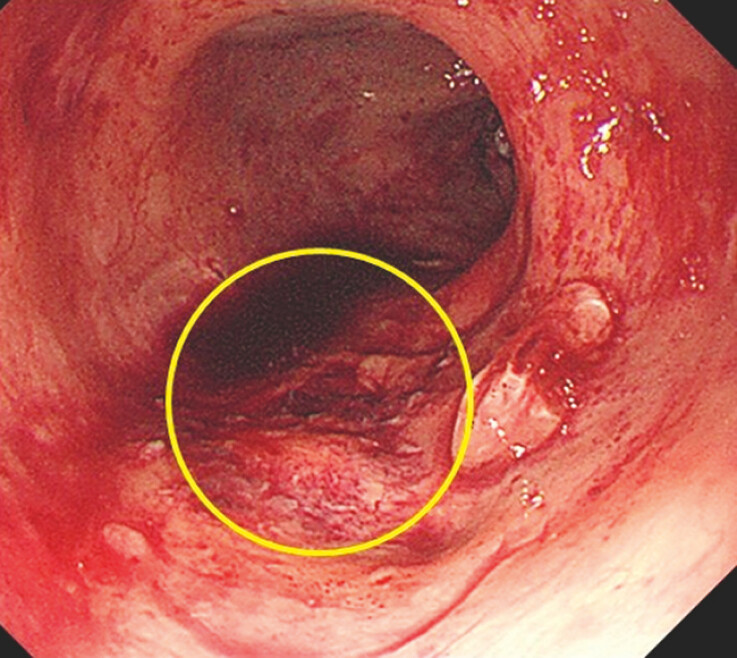
Hemostasis of the varices was complete. The varices themselves also disappeared (yellow circle).

This report presents the first video of cyanoacrylate injection for esophageal varices, resulting in hemostasis and simultaneous disappearance of the varices. This treatment was completed in only one session.Video 1

Treatments including placement of self-expanding metal stents and transjugular intrahepatic portosystemic shunts are options for bleeding esophageal varices that defy application of standard endoscopic treatments such as EVL 4 . Injecting cyanoacrylate into esophageal varices carries a risk of the injectate leaking into the systemic circulation or pulmonary veins via perforating veins 5 or portopulmonary venous anastomosis. However, after confirmation via 3D-CT and/or endoscopic ultrasound that no such collateral circulation has developed, treatment can be administered as in this case. Cyanoacrylate injection for esophageal varices has effects that combine local blood flow blocking and blood flow control, including the blood supply route. This treatment is a useful option for refractory hemorrhagic esophageal varices.

Endoscopy_UCTN_Code_CCL_1AB_2AC_3AG

## Notes

Endoscopy E-Videos https://eref.thieme.de/e-videos
*E-Videos* is an open access online section of the journal *Endoscopy* , reporting on interesting cases and new techniques in gastroenterological endoscopy. All papers include a high-quality video and are published with a Creative Commons CC-BY license. Endoscopy E-Videos qualify for HINARI discounts and waivers and eligibility is automatically checked during the submission process. We grant 100% waivers to articles whose corresponding authors are based in Group A countries and 50% waivers to those who are based in Group B countries as classified by Research4Life (see: https://www.research4life.org/access/eligibility/ ). This section has its own submission website at https://mc.manuscriptcentral.com/e-videos .
